# Castration alters the ileum microbiota of Holstein bulls and promotes beef flavor compounds

**DOI:** 10.1186/s12864-024-10272-8

**Published:** 2024-04-29

**Authors:** Jinping Shi, Zemin Li, Li Jia, Yue Ma, Yongliang Huang, Pengjia He, Tao Ran, Wangjing Liu, Wangdong Zhang, Qiang Cheng, Zhao Zhang, Zhaomin Lei

**Affiliations:** 1 College of Animal Science and Technology, Gansu Agricultural University, Lanzhou, 730070 China; 2 College of Animal Sciences and Technology, Shandong Agricultural University, Taian, 271018 China; 3 College of Pastoral Agriculture Science and Technology, Lanzhou University, Lanzhou, 730000 China; 4 College of Animal Medicine, Gansu Agricultural University, Lanzhou, 730070 China; 5 Gansu Xukang Food Co., Ltd, Pingliang, 744300 China; 6 Gansu Huarui Agriculture Co., Ltd, Zhangye, 734500 China

**Keywords:** Bull, Castration, Slaughter performance, Beef flavor, Ileum, Microorganisms, Metabolites

## Abstract

**Background:**

In the beef industry, bull calves are usually castrated to improve flavor and meat quality; however, this can reduce their growth and slaughter performance. The gut microbiota is known to exert a significant influence on growth and slaughter performance. However, there is a paucity of research investigating the impact of castration on gut microbiota composition and its subsequent effects on slaughter performance and meat flavor.

**Result:**

The objective of this study was to examine the processes via which castration hinders slaughter productivity and enhances meat quality. Bull and castrated calves were maintained under the same management conditions, and at slaughter, meat quality was assessed, and ileum and epithelial tissue samples were obtained. The research employed metagenomic sequencing and non-targeted metabolomics techniques to investigate the makeup of the microbiota and identify differential metabolites. The findings of this study revealed the Carcass weight and eye muscle area /carcass weight in the bull group were significantly higher than those in the steer group. There were no significant differences in the length, width, and crypt depth of the ileum villi between the two groups. A total of 53 flavor compounds were identified in the two groups of beef, of which 16 were significantly higher in the steer group than in the bull group, and 5 were significantly higher in the bull group than in the steer group. In addition, bacteria, Eukaryota, and virus species were significantly separated between the two groups. The lipid metabolism pathways of α-linolenic acid, linoleic acid, and unsaturated fatty acids were significantly enriched in the Steers group. Compared with the steer group, the organic system pathway is significantly enriched in the bull group. The study also found that five metabolites (LPC (0:0/20:3), LPC (20:3/0:0), LPE (0:0/22:5), LPE (22:5/0:0), D-Mannosamine), and three species (*s_Cloning_vector_Hsp70_LexA-HP1*, *s_Bacteroides_Coprophilus_CAG: 333*, and *s_Clostridium_nexile-CAG: 348*) interfere with each other and collectively have a positive impact on the flavor compounds of beef.

**Conclusions:**

These findings provide a basic understanding that under the same management conditions, castration does indeed reduce the slaughter performance of bulls and improve the flavor of beef. Microorganisms and metabolites contribute to these changes through interactions.

**Supplementary Information:**

The online version contains supplementary material available at 10.1186/s12864-024-10272-8.

## Introduction

During beef cattle production, bulls are usually castrated to improve beef flavor and meat quality. In addition, castration can reduce the aggressive behavior of bulls, making production management easier [1]; however, many studies have shown that castration reduces growth and slaughter performance [2, 3]. The decrease in testosterone and androgens can suppress appetite and reduce feed intake, thereby reducing the growth and slaughter performance of bulls [4, 5]. Although a decrease in testosterone can attenuate muscle growth and reduce growth and slaughter performance, it can increase fat deposition [6]. The deposition of fat in muscles directly affects various factors related to meat quality, including tenderness, nutrition, and flavor [7]. Previous studies have focused on the meat quality of castrated beef carcasses [8, 9].

Studies have demonstrated the significant impact of gut microbiota on development and slaughter performance [1]. Castration can alter the gut microbiota, but there are few reports investigating whether this change affects slaughter performance and meat flavor compounds. The prevailing consensus is that the rumen functions as a microbial reactor, facilitating the microbial fermentation of a majority of nutrients [10]. So, research on the small intestinal microbiota is often overlooked. The small intestine serves as the primary organ responsible for the process of digestion and absorption of feed nutrients. It is comprised of three distinct sections, namely the duodenum, jejunum, and ileum. The ileum has a more diverse composition of microorganisms than the anterior segment of the small intestine [11]. It is considered a transitional area before entering the hindgut and contains a higher number of microorganisms, with the number of bacteria remaining above 10% per gram of chyme [12]. The ileum is also a target organ for various drugs, vaccines, nutrients, microorganisms, and metabolites. Changes in the ileum microbiota-host interaction pattern can alter the effective supply of nutrients to peripheral tissues, thereby affecting host metabolism, physiological function, and growth and development [13–15]. In addition, gut microbiota affect the metabolic processes related to fat deposition [16], thereby promoting growth and affecting meat quality. Although the study of the gut microbiota in ruminants has not received much attention, this information is crucial for elucidating the function of the ruminant ileum.

This study aimed to examine the potential effects of castration on the gut microbiota composition in Holstein bulls. Additionally, the alterations in the ileal microbiome were examined to determine whether they influence slaughter performance and beef flavor. The objective of this study was to investigate the underlying mechanism responsible for the alterations in beef slaughter performance and flavor resulting from castration. The research was conducted by examining the microbial composition and metabolite profiles through the application of metagenomic sequencing and metabolomics techniques. The present study offers a comprehensive framework for enhancing beef growth performance and meat quality, both in theoretical and practical aspects.

## Materials and methods

### Experimental model details

The study was carried out at Huarui Pasture, located in Minle County, Zhangye City, Gansu Province. A total of eighteen Holstein bulls were chosen for the experiment, with nine bulls assigned to the bull group (341.41 ± 4.04 kg) and nine 2-month-old Holstein bulls that had been castrated assigned to the steer group (345.13 ± 6.89 kg). Each animal is kept separately in a fence for the duration of the study. There was no statistically significant difference in the starting weight between the two groups, as shown by a *p*-value greater than 0.05. The study consisted of two distinct phases: a 30-day adaptation stage followed by a 270-day testing period. The bovine animals were provided with two daily feedings at 08:00 and 16:00. The bovine animals were provided with two daily feedings at 08:00 and 16:00. The cattle were fed a total mixed ration consisting of corn silage and a grain mixture (Table S1) to meet the Nutrient Requirement of Beef Cattle, 8th Revised Edition, by the Committee on Nutrient Requirements of Beef Cattle and the National Research Council (2016). Throughout the duration of the experiment, unrestricted availability of both feed and water was provided to all study animals. The duration of the fattening experiment was a period of 270 days, remove the individuals with the highest and lowest live weight from each group, and exclude individuals with consistent health conditions lower than other animals. Finally, select 6 animals from each group for slaughter. Sample collection and processing.

On the 270th day of the experiment, all animals involved in the study had a 12-hour fasting period before being euthanized in strict adherence to the guidelines set out by the Animal Welfare and Ethics Committee of Gansu Agricultural University. Following the process of slaughter, several measurements and calculations were conducted in accordance with the methodologies outlined by Keane and Allen [17]. These included the assessment of dressing percentage, carcass weight, eye muscle area /carcass weight, and meat-to-bone ratio (6 per group). A volume of five milliliters of a combination of liquid and solid components was obtained from the ileum of each experimental animal. The collected samples were then transferred to sterile tubes and snap-frozen using liquid nitrogen. Subsequently, the frozen samples were brought to the laboratory on the same day and maintained at a temperature of -80 °C. These samples were intended for metagenomic and metabolomic analysis. A tissue specimen of the ileum, measuring about 2 × 2 cm, was carefully prepared to prevent any compression and subsequently preserved in a 4% paraformaldehyde solution for the purpose of conducting histological investigation. A quantity of around 500 g of the *longissimus lumborum* (the longissimus dorsi between the 12th and 13th ribs of the left half of the carcass) was utilized in order to ascertain the presence and composition of taste components. The sample was collected on March 5, 2021.

### Intestinal morphology

The intestinal samples were extracted from the fixative solution containing 4% paraformaldehyde. The fixed ileum tissues were dehydrated in ethanol, cleared in xylene. Subsequently, the samples were fixed in paraffin and cut into sections measuring 3 μm. These sections were then stained with hematoxylin-eosin staining (H&E) in order to facilitate analysis. Measurements were taken for the height and width of the villi, as well as the depth of the crypts. A total of ten villi, which were fully developed and properly aligned, together with their corresponding crypts from each segment [18], were examined under a Motic BA 210 light microscope (Xiamen, China) at a magnification of 40 ×. The acquired images were subsequently processed using Image-Pro Plus 6.0 software (Media Cybernetics, Rockville, MD, USA).

### Volatile flavor compound analysis

The analysis of volatile flavor compounds in the *longissimus lumborum* meat samples was conducted GC–IMS (FlavourSpec; GAS, Dortmund, Germany). The GC–IMS system was equipped with an MXT-5 capillary column (Restek, PA, USA) and an autosampler (CTC Analytics AG, Zwingen, Switzerland) that included a headspace (HS) sampling unit and a gas-tight syringe (Gerstel GmbH, Mühlheim, Germany). In summary, the ground beef samples were subjected to a thawing process lasting 12 h at a temperature of 4℃. Subsequently, 3 g of the sample was carefully transferred into a 20 mL HS vial equipped with a magnetic screw seal cap (HM-2075G, Hamag Ningbo, Zhejiang, China). The vial was then subjected to an incubation period of 15 min at a temperature of 60℃. Subsequently, an automated injection of 500 µL of hydrogen sulfide gas was performed utilizing an injector temperature of 70℃ and employing a splitless injection technique. The temperature of the column was maintained at 60 °C, while the drift tube temperature was set at 45 °C. The drift gas employed in this study was nitrogen, which was maintained at a flow rate of 150 mL/min. The initial carrier gas flow rate was established at 2 mL/min for a duration of 2 min, with this process being repeated once. Subsequently, the flow rate was elevated to 100 mL/min for a period of 16 min, following which the flow rate was terminated. The sample analyses were performed in triplicate. The retention index (RI) was determined by employing n-ketones C4–C9 (obtained from Sinopharm Chemical Reagent Beijing Co., Ltd., Beijing, China) as external reference standards. The identification of volatile flavor compounds was conducted by the comparison of the retention index (RI) and drift time (Dt) of the library standards in the GC-IMS system. The determination of volatile flavor components was conducted by quantifying the peak heights of the observed signal peaks.

### DNA extraction, library construction, and metagenomic sequencing

The extraction of total DNA from the ileal contents was performed using an OMG-soil kit in Carlsbad, CA, USA. according to manufacturer’s instructions. he concentration and purity of the samples were assessed using a TBS-380 (Promega, Madison, WI, USA) and a NanoDrop™ 2000 (Thermo Fisher Scientific, Waltham, MA, USA). DNA extract quality was checked on 1% agarose gel.

DNA extract was fragmented to an average size of about 400 bp using Covaris M220 (Gene Company Limited, China) for paired-end library construction. Paired-end library was constructed using NEXTFLEX Rapid DNA-Seq (Bioo Scientific, Austin, TX, USA). Adapters containing the full complement of sequencing primer hybridization sites were ligated to the blunt-end of fragments. Paired-end sequencing was performed on Illumina NovaSeq (Illumina Inc., San Diego, CA, USA) at Majorbio Bio-Pharm Technology Co., Ltd. (Shanghai, China) using NovaSeq 6000 S4 Reagent Kit v1.5 (300 cycles) according to the manufacturer’s instructions.

### Sequence quality control and genome assembly

The data were analyzed on the free online platform of Majorbio Cloud Platform. Briefly, the paired-end Illumina reads were trimmed of adaptors, and low-quality reads (length < 50 bp or with a quality value < 20 or having N bases) were removed by fastp [19]. Metagenomics data were assembled using Utilizing Megahit [20]. Using the succeed de Bruijn graph method, the concatenation parameters are iteratively concatenated from small k-mers to large k-mers. Contigs with with a length ≥ 300 bp were selected as the final assembling result, and then the contigs were used for further gene prediction and annotation.

### Gene prediction, taxonomy, and functional annotation

Open reading frames (ORFs) from each assembled contig were predicted using Prodigal/MetaGene [21]. The predicted ORFs with a length ≥ 100 bp were retrieved and translated into amino acid sequences using the NCBI translation table. A non-redundant gene catalog was constructed using CD-HIT [22] with 90% sequence identity and 90% coverage. High-quality reads were aligned to the non-redundant gene catalogs to calculate gene abundance with 95% identity using SOAPaligner [23]. Taxonomic assessment of ileum microbiota was performed using DIAMOND against the RefSeq database. Taxonomic profiles were conducted at domain, phylum, genus and species levels, with relative abundances calculated. The PCoA based on Bray-Curtis dissimilarity matrices at species level was performed. Microbial taxa with a relative abundance > 0.1% in at least 50% of animals within each group were used for downstream analysis [10]. Also, linear discriminant analysis (LDA) with effect size (LEfSe) was performed to identify the important differential microbes between the two groups. Representative sequences of non-redundant gene catalog were aligned to NR database with an e-value cutoff of 1e-5 using Diamond [24] for taxonomic annotations. The KEGG annotation was conducted using Diamond [24] against the Kyoto Encyclopedia of Genes and Genomes database with an e-value cutoff of 1e-5.

### Metabolomic sequencing and bioinformatics analysis

The contents of the ileum were thawed by keeping on ice and agitated for a duration of 10 s. A total of 50 µL of ileum sample content was combined with 150 µL of pre-cooled methanol, which included 1 µg/mL of 2-chlorophenylalanine as an internal standard. The mixture was vigorously shaken for a duration of 3 min and thereafter subjected to centrifugation at a speed of 12,000 rpm at a temperature of 4 °C for a duration of 10 min. The liquid above the solid material was separated and subjected to centrifugation at a speed of 12,000 rpm for an additional 5 min at a temperature of 4 °C. The resulting supernatant was then transferred to a 2 mL container for further analysis using liquid chromatography-tandem mass spectrometry (LC-MS/MS). The metabolome of ileum contents [25, 26] was analyzed using Ultra Performance Liquid Chromatography (UPLC) and MS/MS (QTRAP®). The analysis was conducted using a Waters ACQUITY UPLC HSS T3 C18 chromatographic column with dimensions of 1.8 μm × 2.1 mm × 100 mm. The column temperature was maintained at 40 °C, and the flow rate was set at 0.4 mL/min. An injection volume of 2 μm/min was used for the analysis. The mobile phase was composed of eluent A, which consisted of water containing 0.1% formic acid, and eluent B, which consisted of acetonitrile containing 0.1% formic acid. The gradient elution conditions were as follows: at 0 min, the solvent ratio was 95:5 (v/v); at 10 min, the solvent ratio was 10:90 (v/v); at 11 min, the solvent ratio remained at 10:90 (v/v); at 11.1 min, the solvent ratio reverted to 95:5 (v/v); and at 14 min, the solvent ratio returned to 95:5 (v/v) [27]. The relative concentration of ileum metabolites was analyzed to identify differential metabolites using screening criteria based on FC ≥ 2, FC ≤ 0.5, and VIP ≥ 1. The metabolites that were found underwent annotation using the KEGG compound database (http://www.kegg.jp/kegg/compound/) and the KEGG pathway database.

### Data statistics and analysis

The data underwent statistical analysis to determine significance and Pearson’s correlation using SPSS software version 22.0 (SPSS Inc., IBM Corp., Armonk, NY, USA). The data is provided in the form of mean ± standard deviation (SD). Abundance calculation: Using SOAPaligner software, compare the high-quality reads of each sample with a non-redundant gene set (default parameter: 95% identity), and calculate the abundance information of genes in the corresponding samples. The gene abundance calculation method is Reads Number-Relative: gene abundance is represented by the proportion of the number of reads contained in the gene to all reads in the sample; Calculation formula: $Genei=Ri/{\sum }_{1}^{n}\left(\text{R}\text{i}\right)$, where Ri represents the abundance value of Genei in a certain sample, that is, the number of Reads aligned to Genei in that sample; Represents the total number of reads corresponding to all genes in the sample. Determine whether there is a difference in the distribution of the two groups of populations through Wilcoxon signed-rank test. In total, 865 ileum metabolites were identified and were transformed to have a zero mean and a unit variance for downstream analysis. The study employed OPLS-DA to ascertain the metabolic disparities between the two groups. The OmicShare Tools platform, accessible at https://www.omicshare.com/tools, was used for the execution of two-way O2PLS analysis. Statistical maps were generated using OriginPro 9.1 software (OriginLab, Northampton, MA, USA).

## Results

### Castration reduces Slaughter Performance

The slaughter performance of the bull and steer groups is shown in Table 1. The findings indicate that the carcass weight and eye muscle area /carcass weight of the bull group exhibited a statistically significant increase compared to the steer group (*P* < 0.05). However, no significant differences were seen in the other indicators (*p* > 0.05). This indicates that castration reduces the slaughter performance of Holstein bulls.

**Table 1:** Castration reduces the slaughter performance of Holstein bulls

Items	Bulls	Steers	*P* Value
Dressing percentage (%)	58.45 ± 0.66	57.36 ± 0.47	0.212
Carcass weight (kg)	378.08 ± 7.97	331.14 ± 10.24	0.005
Meat-bone ratio	5.15 ± 0.12	5.15 ± 0.09	0.983
Eye muscle area /carcass weight (cm^2^/kg)	0.22 ± 0.018	0.20 ± 0.012	0.044

### Castration did not alter the ileum epithelium parameters

**Fig. 1:**
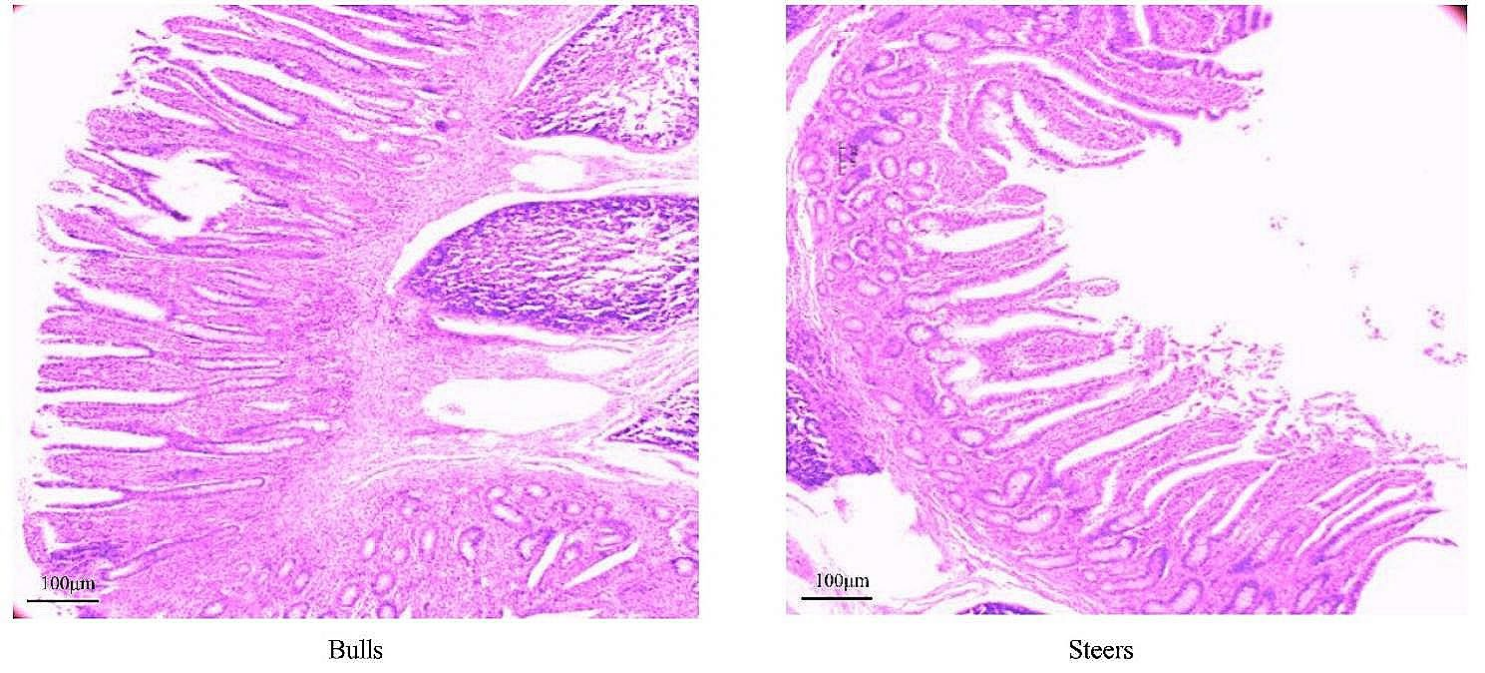
Hematoxylin and eosin staining of ileum papillae

Through H&E staining, we observed the histological morphology of the ileum (Fig. 1). The dimensions of the ileum villi, including length, width, and crypt depth, were shown to be larger in the bull group compared to the steer group. However, statistical analysis indicated that these differences were not statistically significant (*p* > 0.05; Table 2).

**Table 2:** The effect of castration on the morphology of ileum epithelial tissue

Items	Bulls	Steers	*P* value
Villus height(µm)	545.05 ± 11.98b	541.35 ± 9.19c	0.44
Fluff width(µm)	124.21 ± 6.00	117.77 ± 4.98c	0.18
Crypt depth(µm)	278.55 ± 9.33	278.03 ± 14.55	0.11

### Castration improves beef flavor compounds

**Fig. 2:**
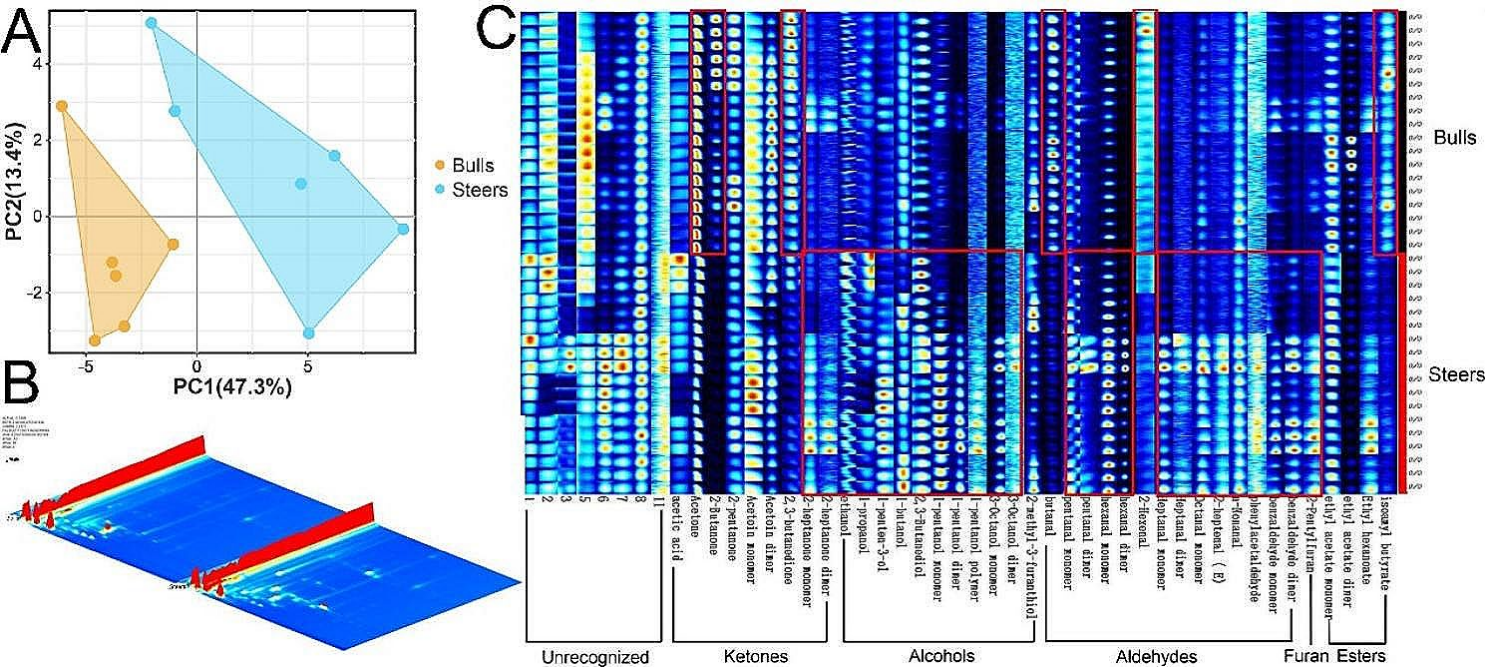
**A:** Analysis of flavor compounds by PCA. **B:** Three-dimensional topographic plots from the bull and steer samples. **C:** Gallery plot from the bull and steer samples

The gas chromatography-ion mobility spectrometry (GC-IMS) technique was employed to identify a total of 53 compounds, out of which 42 were identified correctly by a library search using GC-IMS. A total of 11 ketones, 11 alcohols, 15 aldehydes, four esters, and one furan were identified (Table 3). Principal component analysis (PCA) (Fig. 2A) and three-dimensional topographical plots (Fig. 2B) showed that castration caused significant changes in volatile flavor compounds. The fingerprints showed that castration significantly altered the ketone, alcohol, and aldehyde contents between the two groups (Fig. 2C). In the steer group, the levels of six alcohols, 1-pentanol dimer, 1-pentanol monomer, 1-penten-3-ol, 2,3-butanediol, 3-octanol monomer, and ethanol; two ketones, 2-heptanone monomer and 2-hexanone; seven aldehydes, 2-heptenal (E), benzaldehyde dimer, heptanal monomer, hexanal dimer, hexanal monomer, pentanal monomer, and octanal monomer; and one furan, 2-pentylfuran were significantly higher than those in the bull group (*FDR* ≤ 0.1 or *FDR* ≤ 0.05). The concentrations of 2-butanone, acetone, butanal, 2,3-butanedione, and isoamyl butyrate were found to be considerably elevated in the bull group compared to the steer group (*p* < 0.01 or *p* < 0.05).

**Table 3:** Castration changes the flavor compounds of beef

NO	Compound	Retention index	Retention time, s	Drift time, ms	Intensity, V		FDR
					Bulls	Steers	
Alcohols							
1	1-Butanol	672.4	169.627	1.18265	436.8668 ± 20.223	504.8999 ± 81.245	0.525
2	1-Pentanol dimer	778.4	248.34	1.50922	130.5400 ± 28.644	514.3770 ± 133.836	0.0714
3	1-Pentanol monomer	778.4	248.34	1.25598	446.7989 ± 54.633	1020.8251 ± 194.826	0.071
4	1-Pentanol polymer	776.2	246.584	1.81201	29.2888 ± 1.629	33.9501 ± 2.846	0.260
5	1-Penten-3-ol	694	181.027	0.94581	108.8386 ± 10.786	178.4609 ± 24.63	0.071
6	1-Propanol	572.6	126.061	1.11709	293.6302 ± 14.226	372.6812 ± 67.819	0.381
7	2,3-Butanediol	792.9	262.545	1.35706	493.3946 ± 30.133	776.5095 ± 62.626	0.017
8	3-Octanol dimer	991.7	548.7	1.80442	52.2887 ± 3.344	96.2323 ± 4.962	0.458
9	3-Octanol monomer	991.7	548.7	1.41248	388.7732 ± 79.423	1445.3417 ± 347.487	0.071
10	Ethanol	530.3	107.575	1.04304	7759.5376 ± 420.528	10605.8347 ± 524.536	0.017
11	2-Methyl-3-furanthiol	869.3	346.695	1.14404	1150.2949 ± 216.578	1384.3998 ± 287.855	0.571
Ketones							
12	2,3-Butanedione	585.9	131.854	1.1768	1174.6593 ± 95.518	599.2407 ± 75.767	0.014
13	2-Butanone	590.3	133.798	1.2489	1731.0748 ± 378.655	281.628 ± 11.782	0.046
14	2-Heptanone dimer	892.3	372.162	1.63217	29.7496 ± 2.796	32.7766 ± 3.596	0.5712
15	2-Heptanone monomer	892.8	373.04	1.26149	88.9855 ± 1.823	171.0125 ± 22.055	0.025
16	2-Hexanone	792.9	262.545	1.49883	27.4276 ± 2.268	88.9419 ± 12.823	0.021
17	2-Pentanone	684.5	174.92	1.37644	396.0833 ± 50.705	336.8328 ± 21.583	0.407
18	Acetic acid	622.8	147.955	1.15716	144.595 ± 8.484	172.4957 ± 29.679	0.464
19	Acetoin dimer	732.3	211.563	1.3284	5403.6013 ± 628.065	3904.7121 ± 589.383	0.181
20	Acetoin monomer	734.3	213.192	1.05844	2148.2107 ± 128.083	2237.4722 ± 179.847	0.712
21	Acetone	522	103.939	1.11495	9004.3386 ± 252.470	7009.9177 ± 468.748	0.021
22	Cyclopentanone	797.7	267.898	1.1093	95.4479 ± 9.103	108.5099 ± 22.431	0.631
Aldehydes							
23	2-Hexenal	849.3	324.741	1.17891	102.0048 ± 17.963	82.2967 ± 5.710	0.4074
24	2-Heptenal (E)	958.7	490.143	1.25167	51.1152 ± 2.235	89.6484 ± 10.340	0.023
25	Benzaldehyde dimer	989.8	545.225	1.46599	225.3876 ± 225.387	549.5707 ± 104.611	0.071
26	Benzaldehyde monomer	1002.8	569.551	1.15793	751.5379 ± 126.163	1054.003 ± 113.309	0.180
27	Butanal	598.7	137.463	1.29197	1310.7376 ± 140.947	442.1131 ± 29.194	0.014
28	Heptanal dimer	901.7	388.847	1.6964	26.1585 ± 1.036	36.6709 ± 4.642	0.099
29	Heptanal monomer	902.2	389.726	1.32755	121.3743 ± 8.065	237.663 ± 46.293	0.071
30	Hexanal dimer	795.1	265.025	1.55694	399.287 ± 41.201	1834.4379 ± 508.897	0.074
31	Hexanal monomer	795.9	265.903	1.25415	821.7502 ± 46.047	1722.9628 ± 181.928	0.0214
32	N-Nonanal	1108.7	775.742	1.47226	315.2713 ± 12.981	358.3118 ± 24.888	0.2334
33	Pentanal dimer	695.5	182.249	1.42281	37.6145 ± 2.408	69.8468 ± 19.554	0.233
34	Pentanal monomer	695.5	182.249	1.18762	451.5485 ± 13.655	593.0829 ± 55.869	0.0714
35	Octanal dimer	1012.8	588.964	1.81825	60.8581 ± 2.644	61.7447 ± 2.077	0.797
36	Octanal monomer	1013.3	589.887	1.40724	154.5558 ± 9.557	239.8566 ± 32.966	0.071
37	Phenylacetaldehyde	1043.1	648.072	1.25551	53.9314 ± 2.371	60.24 ± 2.552	0.180
Esters							
38	Ethyl acetate dimer	603.4	139.498	1.33337	964.9372 ± 314.151	402.165 ± 48.631	0.180
39	Ethyl acetate monomer	607.1	141.127	1.09819	708.7041 ± 79.295	764.6409 ± 31.803	0.571
40	Ethyl hexanoate	1008.5	580.652	1.34386	79.4487 ± 9.352	113.3374 ± 17.392	0.182
41	Isoamyl butyrate	1048.1	657.813	1.41269	150.6127 ± 11.015	78.4693 ± 2.976	0.014
Furan							
42	2-Pentylfuran	996.7	557.563	1.24782	52.2887 ± 5.762	96.2323 ± 16.637	0.0714

### Genome profiling of ileum microorganisms

**Fig. 3:**
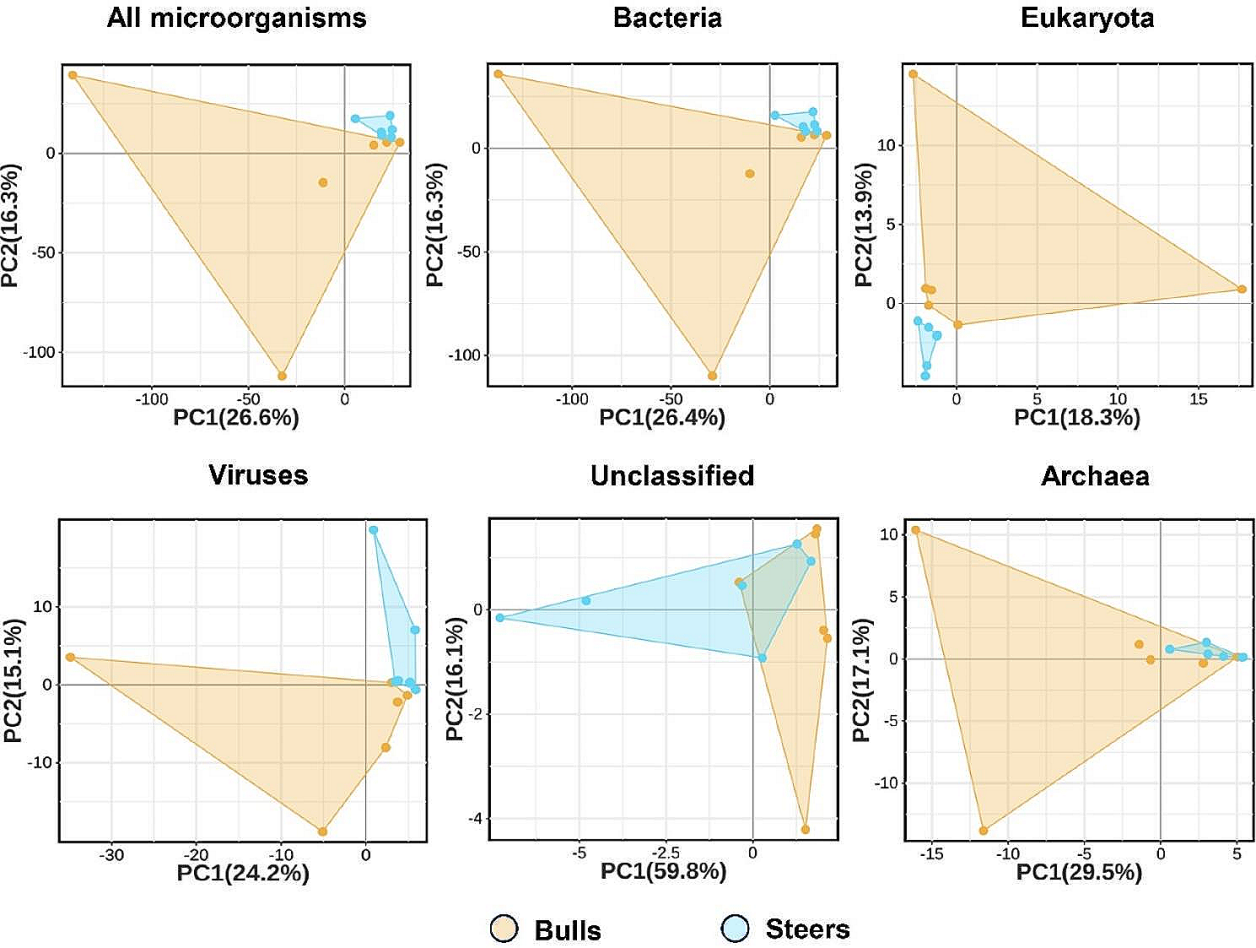
Domain level PCA

Metagenomic analysis was conducted on the ileum contents of the bull and steer groups, resulting in an average of 64,141,039 ± 2,323,322 and 68,294,088 ± 3,254,590 raw reads, respectively. After removing low-quality and unknown reads and host genome sequences, the optimized reads obtained for subsequent analyses were 62,940,063 and 67,095,184, respectively. The proportion of optimized reads, which accounted for 97.82% and 97.85% of the raw reads, suggests that the sequencing outcomes were dependable and suitable for further studies. The principal component analysis (PCA) showed that all microorganisms were separated between the two groups. To further investigate the specific impact of castration on microorganisms, we conducted PCA at the Domain level, which showed that bacterial, eukaryotic, and viral species were separated between the two groups; however, no separation was observed for Archaea or unclassified microorganisms (Fig. 3). Hence, the comparative examination of the microbiota in the ileum of the two groups primarily focused on the identification of bacterial, eukaryotic, and viral components. In addition, the PCA of all microorganisms and bacteria was similar, indicating bacteria were the most abundant ileum microbial kingdom in the ileum of holstein bulls and the differences in the ileum microbial features between bull and steer were mainly found in bacteria.

### Differences in the classification of microorganisms in the ileum

**Fig. 4:**
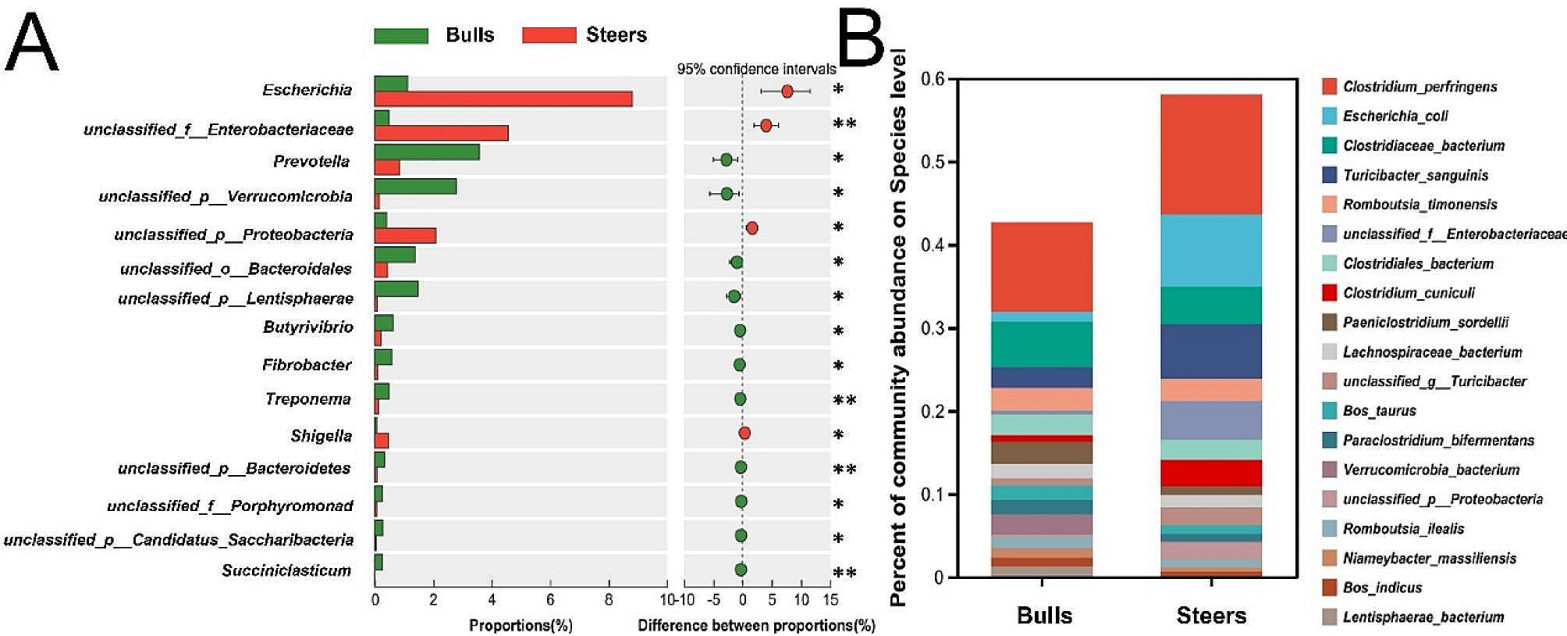
Microbial analysis at the genus and species taxonomic levels. **A:** Differences between the bull and steer groups at the level of genus. **B:** The prevailing strains showed at the species level within the bull and steer groupings

The results of the comparative analysis indicated that three out of the top five genus level exhibited statistically significant variations in abundance between the steer group and the bull group (*p* < 0.05; Fig. 4A): *Escherichia* (bull: 1.12%, steer: 8.81%), *unclassified_F_Enterobacteriaceae* (bull: 0.49%, steer: 4.56%), and *unclassified_ P_ Proteobacteria* (bull: 0.4%, steer: 2.1%). And the abundance of these three genus in the steer group is five times higher than that in the bull group. In terms of species composition (Fig. 4B), the prevailing bacteria seen in the bull group were identified as *Clostridium perfringens* (10.83%), *Clostridiaceae bacterium* (5.51%), and *Romboutsia timonensis* (2.68%). The predominant bacteria in steers were *Clostridium perfringens* (14.42%), *Escherichia coli* (8.69%), and *Turiciactor sanguinis* (6.59%).

A differential analysis was conducted, revealing a total of 393 microorganisms that exhibited significant differences (*p* < 0.05) between the two study groups. In the examination of variations across Archaea populations, it was shown that only seven abundances exhibited statistically significant differences (*p* < 0.05). The comparative study of bacterial diversity revealed a total of 361 bacterial species that exhibited statistically significant differences (*p* < 0.05). Among these, 272 species showed considerably higher abundance in the bull group compared to the steer group, whereas 89 species were found to be significantly more abundant in the steer group compared to the bull group. The comparison of the prevalence of 17 viruses showed statistically significant differences (*p* < 0.05). Specifically, seven viruses had greater prevalence in the bull group compared to the steer group (*p* < 0.05), whereas 10 viruses exhibited considerably higher prevalence in the steer group in comparison to the bull group. A total of six distinct eukaryotic types were observed, with two of them exhibiting considerably more prevalence in the bulls compared to the steer group. Conversely, four eukaryotic types were found to be significantly more abundant in the steer group compared to the bull group (*p* < 0.05; Table S2).

### Functional map and functional differences of ileum microbiome

**Fig. 5:**
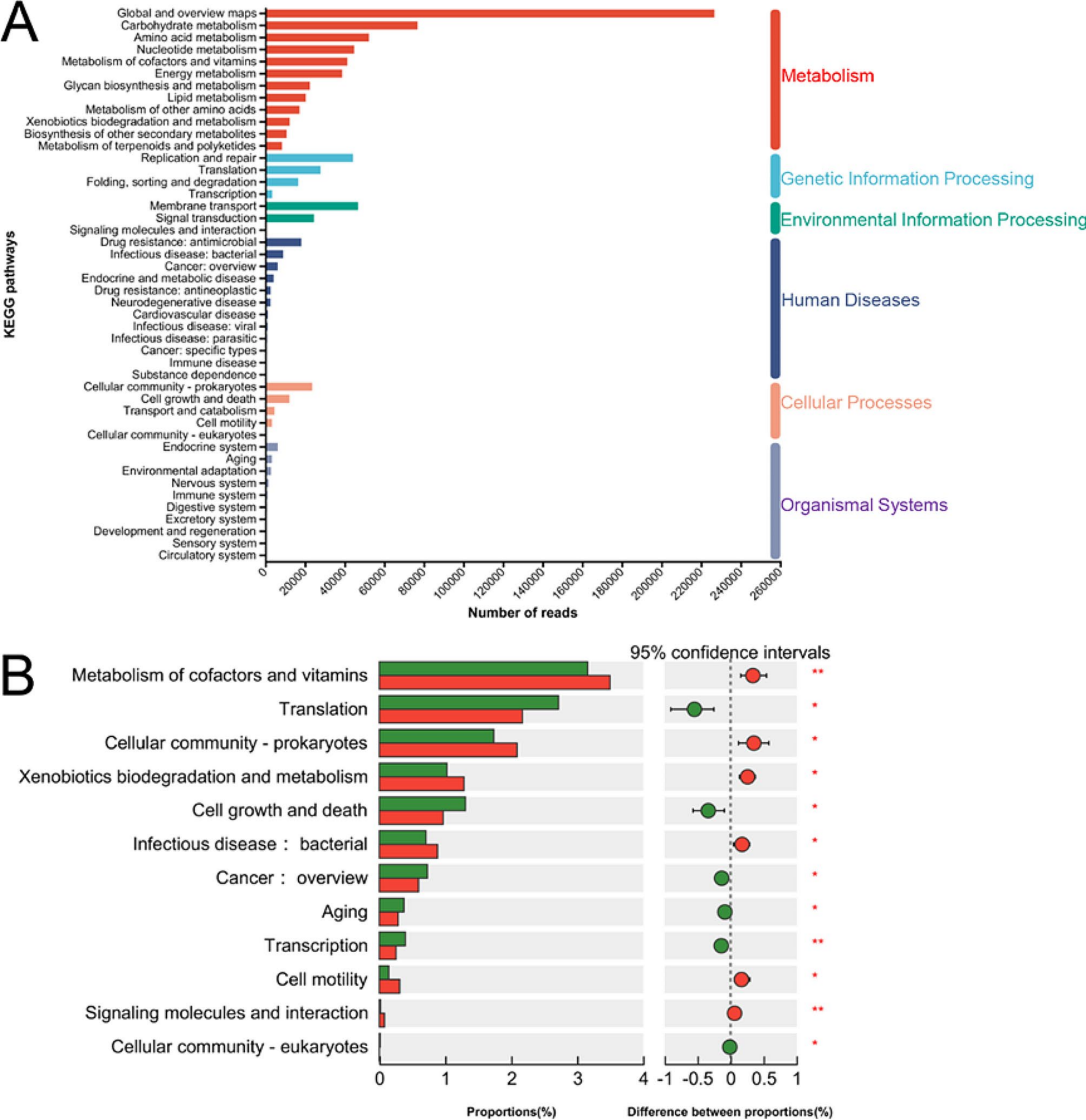
KEGG enrichment analysis. **A:** The enrichment analysis of KEGG pathways at both the first and second levels. **B:** Differentially significant KEGG second-level pathways. **C:** The KEGG third-level pathways had differential significance

For the Kyoto Encyclopedia of Genes and Genomes (KEGG) analysis, a total of six pathways were identified and labeled at the first level. The six major categories of biological phenomena that are commonly studied in academic research are cellular processes, environmental information processing, genetic information processing, metabolism, organizational systems, and human disorders (Fig. 5A).

At the second level of analysis, a total of 46 pathways were, with 12 of these pathways showing substantial enrichment (*p* < 0.05; Fig. 5B). Among the first four pathways with the most abundant enrichment (Metabolism of cofactors and vitamins, Translation, Cellular community prokaryotes, Xenobiotic biodegradation and metabolism), three exhibited substantial enrichments in the group of steers.

**Fig. 6:**
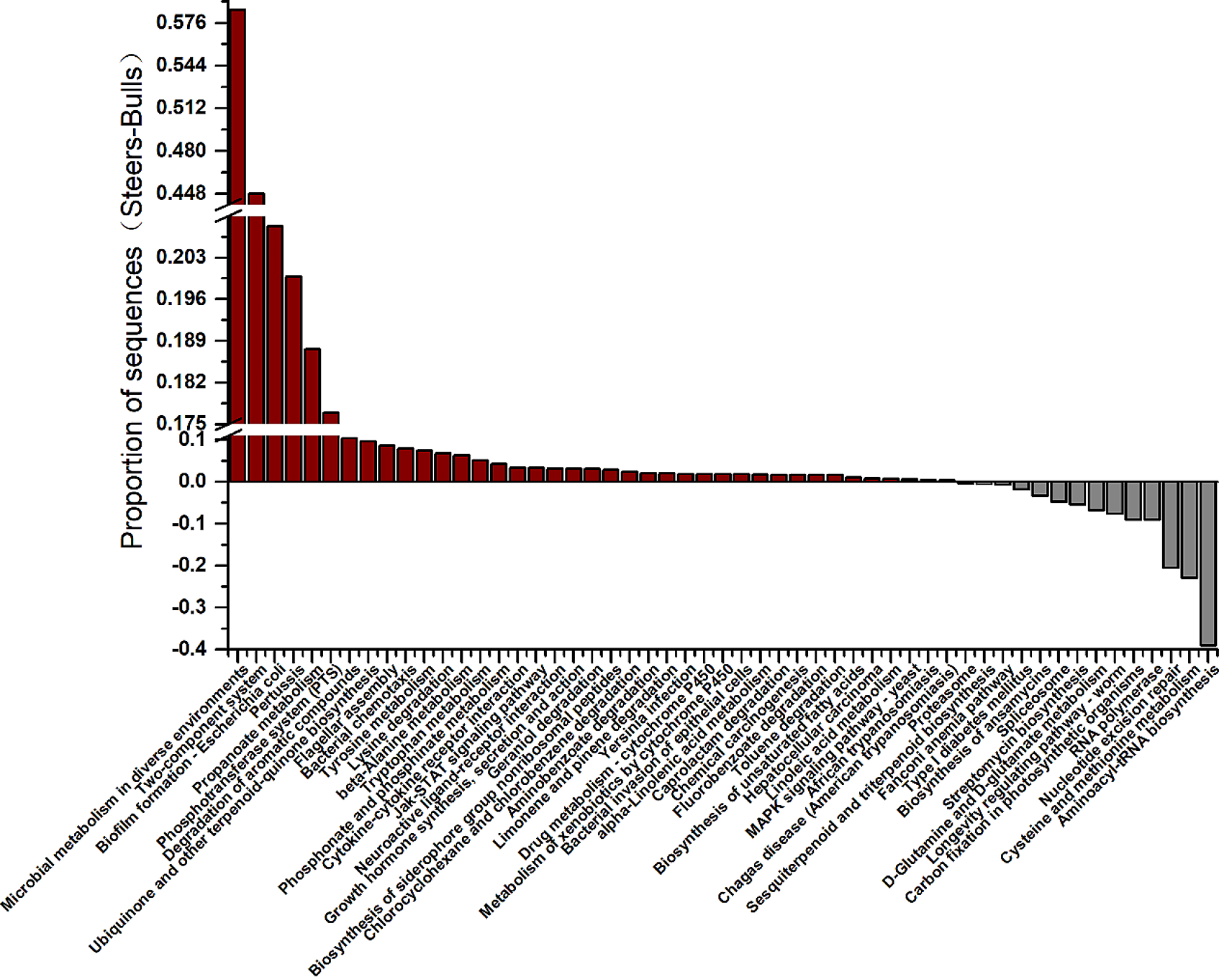
KEGG third-level differentially significant pathways

At the third level, a total of 355 pathways were annotated, of which 53 were significantly different (Fig. 6). Fourteen were significantly enriched in the bull group and 39 were significantly enriched in the steer group. Notably, the Jak STAT signaling pathway and cytokine receptor interaction pathways were only enriched in the steer group.

The carbohydrate-active enzymes (CAZyme) map identified 352 genes encoding CAZymes, including 39 carbohydrate-binding modules (CBMs), 16 carbohydrate esterases (CEs), 11 auxiliary activities (AAs), 55 glycosyltransferases (GTs), 199 glycoside hydrolases (GHs), and 32 polysaccharide lyases (PLs). Only 15 of these genes showed significant differences (Table S3, *p* < 0.05), with 13 significantly enriched in the steer group: three AA, seven GH, two CE, and CBM41, all of which were involved in carbohydrate decomposition. The bull group exhibited substantial enrichment just in the GH50 and GH115 genes.

### Ileum metabolomics analysis

**Fig. 7:**
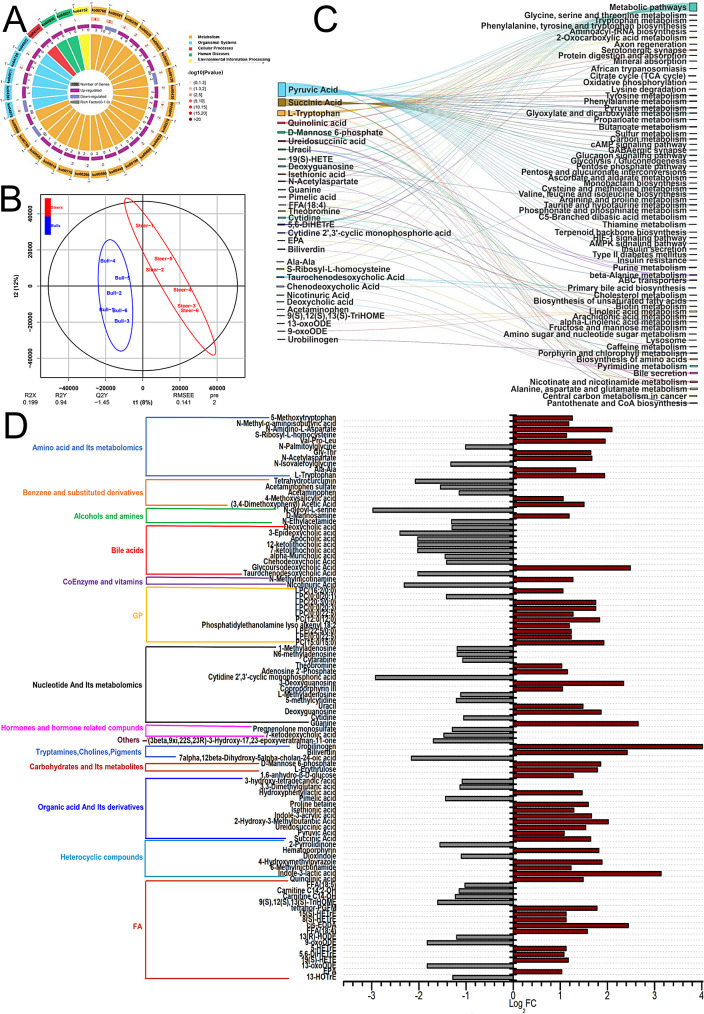
Analysis of metabolites identified in the ileum contents of Holstein bulls. **A:** Enrichment and distribution of metabolites in pathways. **B:** OPLS-DA analysis of the differential metabolites. **B:** Enrichment and distribution of metabolites in KEGG pathways. **C:** KEGG pathways of differential metabolite enrichment. **D:** Differential metabolites between the steer and bull groups

A comprehensive set of 865 compounds were observed in the ileum metabolome. The application of the orthogonal projections to latent structure-discriminant analysis (OPLS-DA) score plot demonstrated the successful discrimination of both groups based on the unique composition of ileum metabolites (Fig. 7B). A total of 98 differential metabolites were found between the bull and steer groups after applying filters based on relative concentrations (fold change [FC] ≥ 2 and FC ≤ 0.5) and variable importance in projection (VIP ≥ 1). Using bulls as controls, the steer group had 57 higher abundance metabolites and 41 lower abundance metabolites (Fig. 7D). These 98 differential metabolites were classified into 17 fatty acids, 14 nucleotides and their metabolomics, 11 organic acids and their derivatives, 11 amino acids and their metabolomics, 10 glycerophorids, nine bile acids, seven heterocyclic compounds, and five benzene and its substituted derivatives. In addition, there were three alcohols and amines; three carbohydrates and their metabolites; three tryptamines, cholines, and pigments; two coenzymes and vitamins; two hormones and hormone-related compounds; and one other. Among the 98 differential metabolites, 31 were enriched through KEGG and were found in 65 pathways (Fig. 7C). The top pathways with the highest levels of metabolite enrichment were Metabolic pathways; Alanine, aspartate and glutamate metabolism; Nicotinate and nicotinamide metabolism; Pyrimidine metabolism; and Bile secretion, which were enriched in 21, 4, 4, and 4 metabolites, respectively. Pyruvic acid was enriched in 34 pathways, and enrichments of succinic acid were observed in 18 pathways (Fig. 7B). The pathways with significant differences in the enrichment of more abundan metabolites were Metabolic pathways (ko01100) and Alanine, aspartate, and glutamate metabolism (ko00250) were enriched in 17 and 4 more abundant metabolites, respectively, and bile secretion (ko04976) was enriched in 4 less abundant metabolites (Fig. 7A).

### Combined metagenome and metabolome analysis

**Fig. 8:**
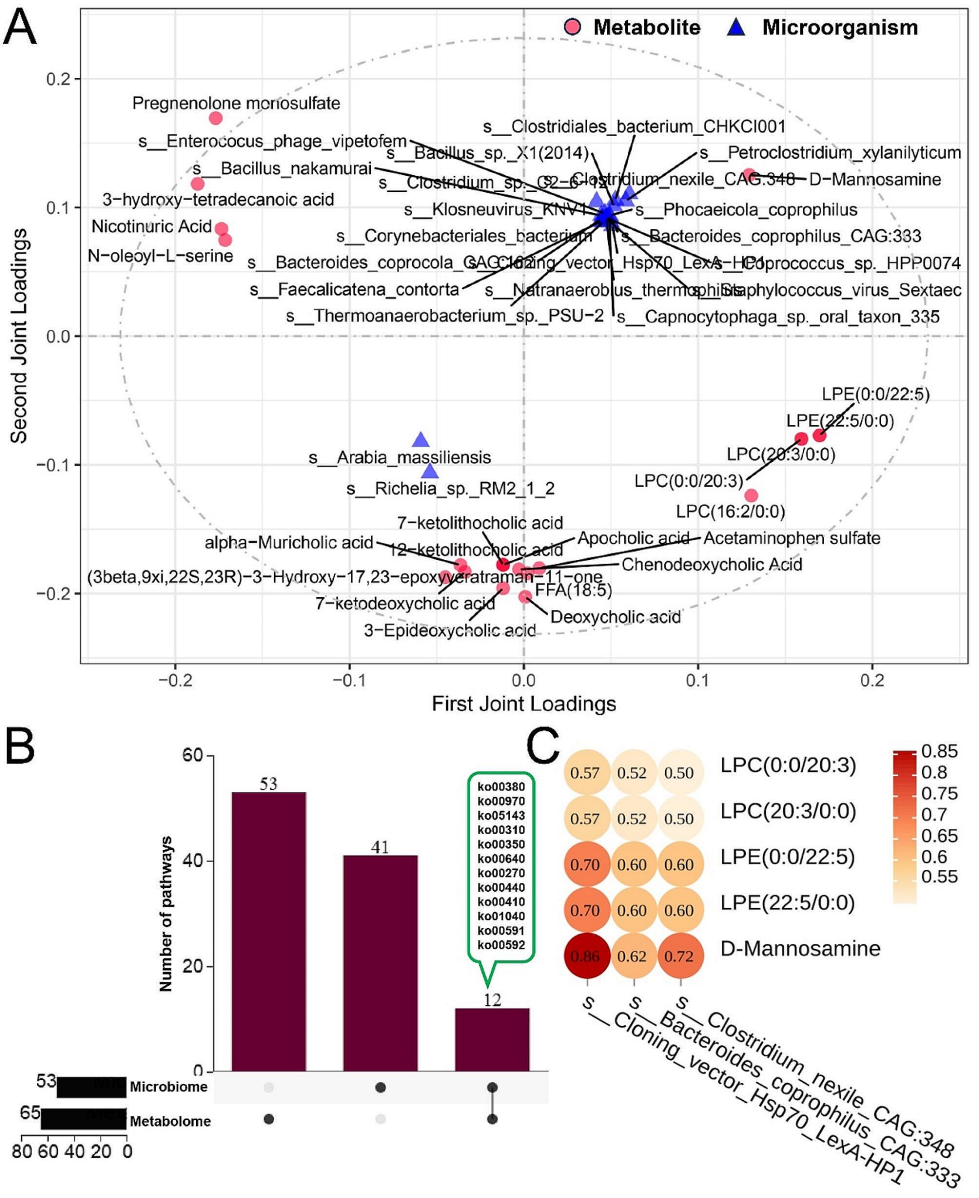
The integration of metagenomic and metabolomic analyses. **A:** O2PLS analyzed metabolomics and macrogenomics. **B:** Venn diagram of KEGG terms co-enriched by differential microorganisms and differential metabolites. **C:** Analysis of the correlation between microbes and the target metabolites

We constructed Venn diagrams for the pathways of differential metabolite and microbial enrichment and found that 12 pathways, including Tyrosine metabolism (Ko00350), Cysteine and methionine metabolism (ko00270), and Tryptophan metabolism (ko00380), were co-enriched in both metabolites and microorganisms (Fig. 8B). Notably, 10 of these 12 common pathways were upregulated in the steer group. To determine whether microorganisms and metabolites have a linkage effect, all microbiome and metabolome data were analyzed, and orthogonal partial least squares (O2PLS) analysis was used to identify the top 20 microbes and metabolites with the strongest linkage effect (Fig. 8A). This indicated a strong interrelationship between microorganisms and metabolites. The top 20 metabolites obtained from O2PLS analysis based on VIP ≥ 2.5 were rigorously screened and ultimately obtained five differential metabolites, LPC (0:0/20:3), LPC (20:3/0:0), LPE (0:0/22:5), LPE (22:5/0:0), and D-Mannosamine; all of which were upregulated. Similarly, we rigorously screened 20 microorganisms with *p* ≤ 0.01 and ultimately obtained three microorganisms, *an unclassified species s_Clonning_vector_Hsp70_LexA-HP1 and two bacteria s_Bacteroides Copihilus-CAG: 333 and s_Clostridium nexile-CAG: 348.* In the steer group, the abundance of these three microbes increased. In order to delve deeper into the association between these five metabolites and three microbes, Spearman correlation analysis was performed. The findings indicated that these five metabolites positively governed three microorganisms, with a strong (ρ ≥ 0.8) and moderate (0.5 ≤ ρ < 0.8) regulatory relationship (ρ ≤ 0.5; Fig. 8C).

### Correlation analysis

**Fig. 9:**
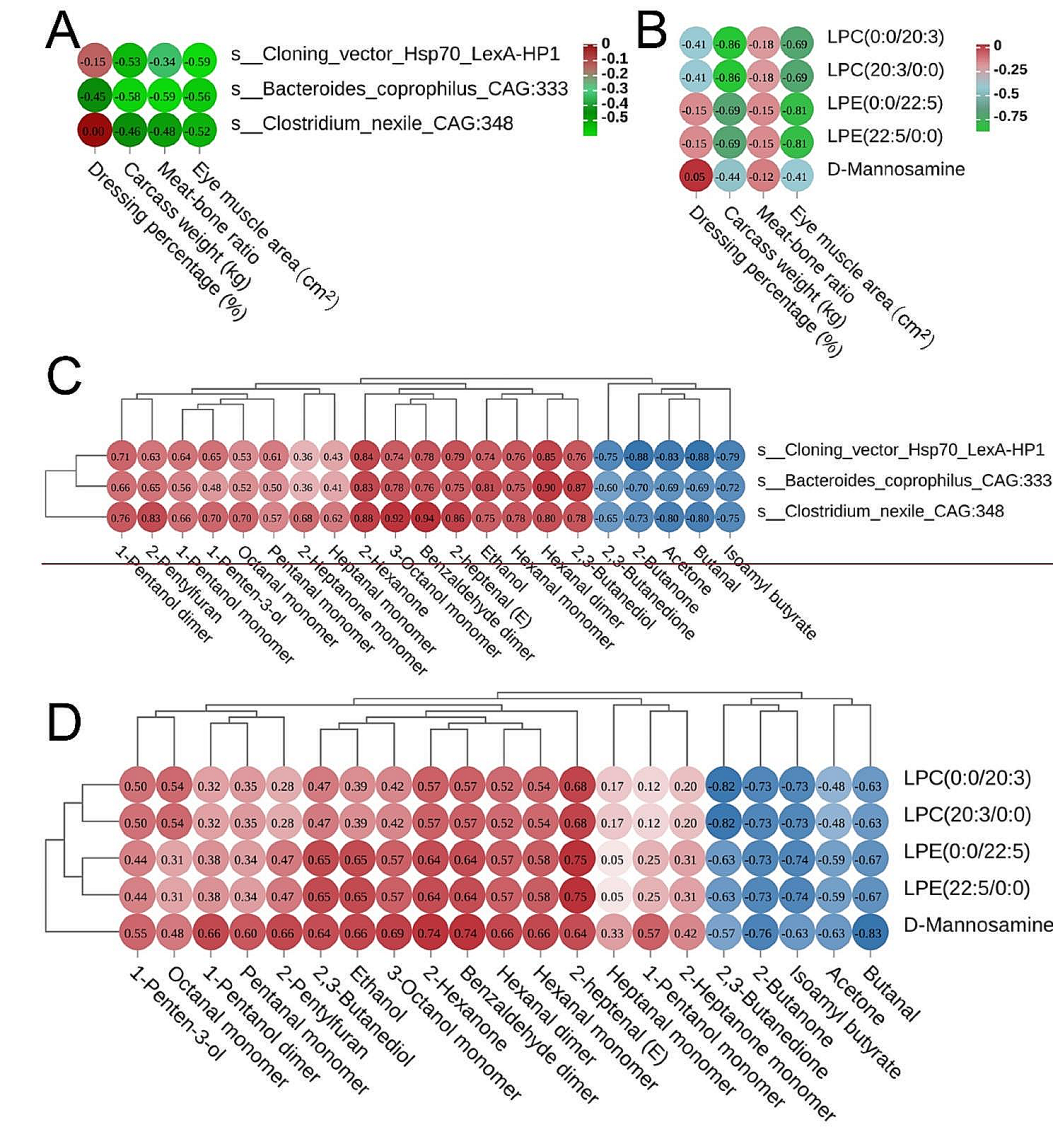
**A:** Correlation between key microorganisms and slaughter performance. **B:** Slaughter performance and key metabolites correlation. **C:** Key microbes and beef flavoring compounds correlation. D: Correlation between key metabolites and beef flavor compounds

In order to investigate the impact of five metabolites and three microorganisms, an analysis was conducted on the slaughter performance and flavoring compounds present in the longissimus lumborum (the longissimus dorsi between the 12th and 13th ribs of the left half of the carcass). The findings indicated that the three microorganisms that exhibited higher abundance demonstrated a negative association with meat-to-bone ratio, dressing, eye muscle area /carcass weight, and carcass weight (Fig. 9A). *s_Bacteroides_Coprophilus_CAG: 333* showed a moderate negative correlation with carcass weight and meat-to-bone ratio (*ρ≤-0.5*). The five most abundant metabolites showed a negative correlation with carcass weight, meat-to-bone ratio, dressing percentage, and eye muscle area /carcass weight (*ρ* < -0.1, Fig. 9B). This indicates that castration resulted in the upregulation of these three microorganisms and five metabolites and that their interaction led to a decrease in slaughter performance. The correlation results for flavor substances showed that the three microorganisms and five metabolites were positively correlated with 16 upregulated flavor compounds (1-Pentanol dimer, 1-Pentanol monomer, 1-Penten-3-ol, 2,3-Butanediol, 2-Heptanone monomer, 2-Hexanone, 2-Pentylfuran, 3-Octanol monomer, Benzaldehyde dimer, Ethanol, Heptanal monomer, Hexanal dimer, Hexanal monomer, Octanal monomer, Pentanal monomer, 2-heptenal (E)) and negatively correlated with five downregulated flavor compounds (2,3-Butanedione, 2-Butanone, Acetone, Butanal, Isoamyl butyrate) (Fig. 9C, D), indicating that castration can increase flavor compounds by altering microorganisms and metabolites.

## Discussion

In recent years, beef quality and flavor have become the most economically significant factors for livestock production, and castration is of great importance in improving beef quality. However, the phenomenon of poor slaughter performance caused by castration remains evident, and the specific mechanism from a microbial perspective remains to be determined. This study analyzed slaughter performance and ileum epithelial tissue morphology to characterize the changes after castration. We demonstrated the important association of the ileum microbiome with ileum metabolites in beef flavor differences using ileum metagenomics and metabolomics to elucidate the mechanisms involved.

Since the year 1990, a number of systematic studies have been conducted that have demonstrated the potential reduction in the growth performance of beef cattle as a result of castration [28]. Although castration reduces growth performance, it has a significant positive effect on meat quality [29], which is widely believed to be due to the reduction in hormone concentrations, particularly testosterone. Owing to differences in testosterone-mediated nitrogen metabolism, a larger deposition of lean tissue leads to weight gain [4]. In line with prior findings, the present study demonstrates that the Dressing percentage and Meat one ratio was no significant difference in, but the carcass weight and eye muscle area /carcass weight of bulls were notably higher in comparison to the steer group. These results suggest that bulls exhibit favorable slaughter performance. Our previous research showed that the serum testosterone, triiodothyronine, thyroid hormone, and growth hormone levels in the steer group were significantly lower than those in the bull group (*P* < 0.05), while the levels of somatostatin were significantly higher than those in the Bull group (*P* < 0.05) [1]. This may be an important reason why the slaughter performance of the bull group is better than that of the steer group.

Prior research has investigated the impacts of castration on meat quality, growth, and slaughter performance [30, 31]; on the other hand, nothing is known about the general modifications to the gut microorganisms of castrated ruminants. In this study, PCA analysis showed that bacteria, Eukaryota, and viruses were isolated in the ileum of the castration and bull groups but no isolates with archaea. The cellulose consumed by ruminants is mainly broken down in the rumen to produce H_2_ and CO_2_, whereas Archaea can synthesize methane from H_2_ and CO_2_, which is of great importance for maintaining normal H_2_ partial pressure in the rumen [32]. However, the ileum barely decomposes cellulose. Therefore, the numbers of Archaea are limited and do not affect the entire microbial community of the ileum. The PCA analysis of bacteria was consistent with that of all microorganisms, indicating that the contribution of bacteria to the ileum is more important than that of other microbial kingdoms. This is in line with findings from a study of bacteria found in ileum of other animals [33]. Our results indicate that castration altered the microbiota of the ileum, both in terms of microbial species and quantity and the predominant bacteria. Prior research has demonstrated that alterations in the microbial composition inside the gastrointestinal tract, resulting from high-precision diets and antibiotics, can damage the intestinal mucosa and cause changes in the intestinal epithelium [34, 35]. A single layer of epithelial cells that make up the small intestinal epithelium is vulnerable to the effects of the digestive tracts microbes and surroundings [36]. The ileal epithelium’s tissue morphology did not significantly differ between the two groups, according to the study’s H&E staining data. According to the outcomes, it can be postulated that the mechanism of microbial alteration in the ileum caused by castration differs from those caused by other factors. Changes in the microbiota caused by castration did not affect the normal function or epithelial morphology of the intestine. The surface area of the gastrointestinal epithelium is important for nutrient absorption [37]. In this study, the differences in crypt depth, villus height, and width in the ileum were not significant, indicating that castration did not reduce slaughter performance by attenuate the absorption rate of nutrients.

The predominant bacteria shared by bulls (10.83%) and steers (14.42%) was *Clostridium perfringens*, which is not conducive to animal growth and development. *Clostridium perfringens* is closely associated with mastitis in cows [38] and is widely present in the small intestine of cattle [39], which may be the result of genetic or environmental factors. Research has indicated a highly significant negative correlation between the quantity of *Clostridium perfringens* in the ileum and an animal’s weight gain [40]. Their higher abundance in the steer group may be one of the reasons for the lower slaughter performance compared to the bull group.

*Turiciactor sanguinis* is involved in carbohydrate fermentation, host lipid metabolism, and short-chain fatty acid synthesis [41, 42] and was one of the predominant bacteria in the steer group. Furthermore, the enhanced JAK-STAT signaling pathway, specifically observed in the steer group, facilitates the modulation of several hormones, hence exerting a significant influence on the maturation and functionality of adipocytes [43]. Carbohydrates, fats, and fatty acids directly affect flavor compounds in meat [44], and these differences may have led to differences in flavor compounds between the two groups.

Raw meat exhibits few olfactory or gustatory attributes and possesses a taste reminiscent of blood. Conversely, cooked meat acquires distinct flavor profiles due to the intricate interplay of flavor precursors present in meat protein and fat constituents. This interaction culminates in the generation of volatile flavor compounds [45]. More than 700 volatile taste compounds have been found, encompassing a wide range of chemical classes such as alcohols, furans, ketones, esters, aldehydes, and pyrazines. Many more compounds are found in beef than in other meats [46]. These compounds, alone or in combination, give beef its unique aroma, flavor, and palatability, thereby affecting consumer acceptance. The oxidative degradation of linoleic acid produces hexanal and glutaraldehyde, which contribute to its subtle aroma [47]. Although n-heptaldehyde can cause a strong fatty and putrid taste [48], octanal and n-heptaldehyde together produce nutty and fruity aromas. After dilution, octanal has a fatty and fruity odor. Benzaldehyde imparts a fragrant aroma to almonds [49] and is mainly characterized by its nutty aroma [50]. Alcohols contribute to the herbaceous, woody, and fatty flavors of meat [51]; for example, 2,3-butanediol has an onion flavor [52]. 2-pentylfuran has a fragrant scent. Although the amount of 2-butanone, acetone, butanal, 2,3-butanedione, and isoamyl butyrate were found to be considerably elevated in the bulls compared to the steer group, the threshold for most ketones was high, and their contribution to flavor characteristics was minimal [53]. For example, extremely low content of 2,3-butanedione results in cheese aroma [54].

The main CAZyme families include GTs, involved in synthesis; CEs, involved in decomposition; GHs, PLs; and CBM [55]. GH, CE, and PL decompose cellulose, hemicellulose, starch, and pectin through synergistic effects [56]. The GH family is mainly composed of starch hydrolases. Starch is hydrolyzed into maltose and glucose and, through the glycolytic pathway of glycolytic bacteria, produces pyruvic acid (intermediate step), which in turn produces metabolites such as volatile fatty acids, CO_2_, and CH_4_. The enrichment of CAZyme genes encoding GH, CE, AA, and CBM involved in carbohydrate degradation in the ileum microbiota of the steer group further demonstrates that their microorganisms have a high carbohydrate degradation ability and may produce more hydrolysates and pyruvic acid. Our metabolomic data confirmed that the steer group had higher levels of pyruvic acid. Pyruvic acid is oxidized in the mitochondria to form acetyl-CoA, which synthesizes fatty acids through the tricarboxylic acid cycle, promotes intramuscular fat deposition [57, 58], and improves meat quality.

Through combined analysis, we found 12 differential pathways enriched by differential microorganisms and metabolites, of which 10 were significantly enriched in the steers group. Out of the 10 pathways examined, it was found that five signaling pathways, namely ko00380, ko00410, ko00310, ko00350, and ko00640, were associated with amino acid metabolism. Additionally, three pathways, including ko00591, ko00592, and ko01040, were found to be involved in the metabolism of linoleic acid, linolenic acid, and unsaturated fatty acids, respectively. Many studies have shown that lipids and amino acids are the main contributors to meat flavor compounds [59], which is consistent with our results.

The organic system pathway was significantly enriched in the bull group and mainly included multiple systems such as immunity, digestion, endocrine, circulation, and growth and development [60]. Enhancements in the organic system yield advantageous outcomes for the physiological maturation and advancement of the human body, which suggests that the bull group may have a higher feed reward and weight gain. The results of this study and our previous research [1] confirm this inference.

The functions of microorganisms in the body are usually achieved through metabolites; therefore, it is important to explore the interactions between microorganisms and metabolites. Through O2PLS analysis, we found a strong mutual relationship between the microorganisms and metabolites. Spearman correlation analysis of the five metabolites and three microorganisms identified that there was a moderate and strong regulatory relationship (ρ> 0.5). Although the correlation analysis cannot indicate a regulatory relationship between them, it can show a certain correlation between them, which is consistent with the results of our O2PLS analysis. The correlation between these three microorganisms and five metabolites, slaughter performance, and flavor compounds indicated that these three microorganisms and five metabolites were negatively correlated with slaughter performance but positively correlated with muscle flavor compounds, which is consistent with the results of our determination of flavor compounds. Studies have shown that feeding LPC to pigs can lead to a decrease in carcass performance but have a positive impact on muscle fat deposition [61]. LPC can also affect the flavor of beef and enhance aftertaste [62]. During heat treatment of beef, some polar lipids (PC) are hydrolyzed into LPC and PLE, which are beneficial for fat deposition in beef and play an important role in improving meat quality [63]. D-mannosamine is only present in a limited subset of bacterial polysaccharides and is specifically located within the cellular wall of Bacillus. Although the biochemical basis of the mechanism remains unclear, it has been shown to have an inhibitory effect on growth, which is achieved by reducing glucose utilization [64]. This may contribute to the lower slaughter performance of the steer group in the current study. Furthermore, it was noted that the three species exhibited higher abundance, *an unclassified species s_Clonning_vector_Hsp70_LexA-HP1 and two bacteria s_Bacteroides Copihilus-CAG: 333 and s_Clostridium non-exile-CAG: 348.*, also played important roles in growth. *Bacteroides copropophilus* is an anaerobic gram-negative bacterium, with the main end products being succinic acid and acetic acid, as well as small amounts of isovaleric acid, propionic acid, and pyruvic acid. Succinate and pyruvic acids participate in the tricarboxylic acid cycle and promote energy metabolism [65]. *Clostridium nexile* can lead to significant differences in metabolites and is positively correlated with muscle growth [66]. *s_Cloning_vector_Hsp70_LexA-HP1* as a newly vector, the role of this organism and related mechanisms are still unclear.

## Conclusion

This work has successfully observed the categorization features, functions, and metabolites of bacteria in the ileum. Additionally, it has examined the relationships between these microorganisms and metabolites with their impact on host growth performance and the production of beef taste-enhancing compounds. The ileum microbiota composition, function, metabolites, and host metabolism of castrated Holsteins were significantly different from those of Holstein bulls. The microorganisms and metabolites of bulls are beneficial for growth and development and have a positive impact on slaughter performance but are not conducive to the flavor of beef. While castrated bulls exhibit reduced slaughter performance, their meat is characterized by enhanced flavor compounds, a quality that is intricately linked to the metabolic processes of microorganisms involved in lipid and amino acid metabolism. This study offers a novel elucidation for the aforementioned phenomena, focusing on the role of microorganisms and metabolites. Through comprehensive analysis, the study has successfully found a total of five metabolites and three microbes that synergistically contribute to the enhancement of taste components in beef.

### Electronic supplementary material

Below is the link to the electronic supplementary material.

Supplementary Material 1

Supplementary Material 2

Supplementary Material 3

## Data Availability

All data generated or analyzed during this study are included in this article, and the raw data can be obtained by contacting the corresponding author. All sequencing data are available through the NCBI Sequence Read Archive (Bio Project ID: PRJNA1033150).

## Abbreviations

CAZYme: Carbohydrate-active enzyme
CBM: Carbohydrate-binding modules
CE: Carbohydrate esterase
DA: Orthogonal Projections to Latent Structures
FC: Fold change
FDR: False discovery rate
GH: glycoside hydrolases
Gt: Glycosyl transferases
KEGG: Kyoto Encyclopedia of Genes and Genomes
NCBI: National Center for Biotechnology Information
O2PLS: Two-way Orthogonal Partial Least Squares
OPLS-DA: Orthogonal Projections to Latent Structures Discrimination Analysis
PL: Polysaccharide lyases
PCoA: Principal coordinates analysis
TMR: Total mixed ration. UPLC: Ultra Performance Liquid Chromatography
VFDB: Virulence Factor Database
VFA: Volatile fatty acid
VIP: Variable importance in the projection
